# Foreign Correspondence

**Published:** 1854-06

**Authors:** Austin Flint

**Affiliations:** Professor of the Theory and Practice of Medicine in the University of Louisville


### MEDICINE AND SURGERY.

JUNE, 1854. NEW SERIES. VOL. I.—NO. 6.

### FOREIGN CORRESPONDENCE.

BY AUSTIN FLINT, M. D., PROFESSOR OF THE THEORY AND PRACTICE OF MEDICINE IN THE UNIVERSITY OF LOUISVILLE.

PARIS, April 19, 1854.

DEAR DOCTOR:—

The Hospitals in Paris are the objects which first engage the attention of the medical stranger. I arrived in the city on Saturday evening, and on Monday morning I found my way to *Hôtel Dieu.* This, the most ancient, and one of the largest hospitals in Paris, first claims attention, and I propose in this letter to give you some account of it, premising that my observations have, as yet, been in a great measure confined to the medical wards, at present under the charge of Prof. TROUSSEAU.

*Hôtel Dieu* was founded in the seventh century. The chief entrance is in the *ile de la cité*, opposite the cathedral of *Notre-dame.* The ancient buildings, several in number, and extensive, are on this island. These are soon to be removed, and a new construction to take their place. A large building, of modern date, is on the opposite side of the southern branch of the river Seine, connected with the ancient portion by a covered bridge, and a tunnel beneath the quay intervening between the river and the building. The latter, situated in the *Enclos Saint Julien* embraces commodious and airy wards with one hundred and four beds. The total number of beds which the hospital contains is eight hundred and ten. Surgical and medical cases are received at this hospital with the exception of children, incurables, insane persons and those affected with cutaneous or syphilitic diseases. For each of the classes of cases just enumerated there are special hospitals. Lying-in patients are only received in cases of emergency. This, together with all the hospitals of Paris, and everything pertaining to public assistance, is under the direction of a body, termed the *administration générale de l'assistance publique à Paris*, consisting of the Minister of the Interior, a Director, and a *Conseil de surveillance*, consisting of twenty members, over which preside the prefects of the Seine and of the police. The yearly average number of patients at *Hôtel Dieu* is twelve thousand, and the mortality one in eight.

The medical staff consists of Drs. LOUIS,* HEURTELOUPE, REQUIN, PIEDAGNEL, ROSTAN, GUERARD, BOUCHET, GRISOLLE, and TROUSSEAU, —Physicians; P. BOYER, JOBERT,—Surgeous. A vacancy in the Surgical department now exists, in consequence of the death of the eminent ROUX.† The duties are distributed among the different medical and surgical officers, so that an undue number of cases are not imposed upon any one. Thus, at the present time, only two wards, one for female, and the other male patients, are assigned to M. TROUSSEAU, the two containing about a hundred beds, and averaging from seventy to eighty inmates. The patients in the hospital are nursed by thirty-three *religieuses* and twelve novices of the order of St. Augustin. To each ward, also, are assigned several *internes* and *externes*, sub-medical officers, and a *pharmacien* or apothecary. The *irternes* and *externes* are appointed by *concours*, and receive a small salary. The medical officers on duty at this, as at other hospitals, are authorized to admit urgent cases; but the distribution of patients among the different hospitals in Paris is managed at the *Bureau central d'admission*, situated near *Hôtel Dieu*. For this duty there is a Board of twelve Physicians and six Surgeons who are on service in rotation.

As stated in my previous letter, M. TROUSSEAU commenced his

* LOUIS has lately resigned, and I know not who is his successor.

† The vacancy has been filled by the appointment of M. RICER.

clinical course a few days after I arrived at Paris. From his writings and his reputation as an oral teacher, I was desirous of seeing his hospital practice, and hearing him lecture, and, as I have already said, my visits at *Hôtel Dieu* have been for the most part confined to his wards and to the amphitheatre at his lecture hour. M. Trousseau was not long since appointed at *Hôtel Dieu* in the place of Chomel, who was compelled to retire in consequence of declining to take the oath of allegiance to the present ruling dynasty of France. I am informed that the acceptance by Trousseau of the position under these circumstances has rendered him in some degree unpopular with the medical profession at Paris. As a clinical teacher in medicine, however, no one, at the present time, is so much esteemed by medical students, judged by the numbers who follow him through the wards, and assemble at his lectures.

In making his morning tour, through the two wards, commencing at a quarter before 8 o'clock, A. M., M. Trousseau occupies an hour and a quarter. This allows him to remain but a few moments at the bed side of most of the patients; and the examination in no instance is much prolonged. No notes are referred to, save that he holds in his hand a sheet containing the prescriptions of medicine, diet, etc., heretofore made, and this is the only record of the history of the case which appears to be made. If daily records of the symptoms are noted, it must be done privately by the *internes*, and are not produced. The age, occupation and time of admission of each patient is affixed to the bed. A space is reserved for the title of the disease, but I have not observed this space filled out anywhere except at the Hospital St. Louis.

All the clinical Professors appear in the wards in hospital costume. This consists of an old overcoat, a white apron, and a close cap of cloth or velvet. M. Trousseau's ward coat, coarse and much the worse for wear, resembles what with us is called a pea-jacket. The red *rosette* of the legion of honor, however, graces the collar. He appears in the amphitheatre and gives his lectures in the dress worn in the wards. The lectures, both clinical and didactic are always given sitting, and generally the lecturer has before him few or no notes.

At the opening of the clinical course by M. Trousseau, about a hundred physicians and students assembled in the wards while the examination of patients was going on. Very little was said at the bed side. The prescription for the day, the diet, &c., constituted the bed side instruction.

The amphitheatre on the first day was crowded. I should judge there were about two hundred persons present, the major part appearing to be medical students. At the subsequent lectures the attendance has been less, varying fron one hundred to one hundred and fifty persons. The utmost order, both here and elsewhere, in the lecture room is preserved. The students generally evince close attention, and many take notes. As regards a general aspect of intelligence and cultivation, however, the French students do not appear to me to be superior to medical classes in the United States. I have not noticed any impropriety of deportment anywhere, save that persons are in the habit of dropping in, and going out irregularly while the lectures are going on.

The lecturers here are not, so far as I have observed, often greeted at their entrance with much applause, and at the conclusion of the lecture demonstrations of approbation are slight, or altogether wanting. None are made during the progress of the lecture. In these particulars a marked difference exists between the customs here and with us. I am led to notice this the more because from the enthusiasm and vivacity for which the French people are distinguished, one would not naturally expect so much quiet in the lecture room.

M. TROUSSEAU appears to be about fifty years of age. He is tall, rather spare in figure, his black hair interspersed with a few that have become grey, is arranged so as to conceal, without close inspection some deficiency on the crown. His physiognomy denotes quickness and sharpness of intellect rather than a meditative, profound mind. The expression of his countenance is pleasing. His voice is agreeable and his intercourse with the patients and nurses is pleasant and kind. He is also affable in his relations to the *internes* and the students, at the same time always preserving a dignified demeanor.

As a lecturer he is admirable. His enunciation is remarkably distinct, so that he may be followed in his discourse by one imperfectly acquainted with the French tongue. He speaks with sufficient deliberation, but with the utmost fluency, making not the least attempt either at rhetorical or oratorical display, entering into his subject with interest, and often manifesting considerable animation. In confining himself to a strictly didactic colloquial style, he follows the uniform custom with the public lecturers here, so far as I have yet observed. On this point, however, it is to be remarked that, if I may presume to say it, the French language is much less adapted to oratory and rhetorical power than our own. It does not admit of the rounded, ponderous sentences, and the *ore rotundo* which give to English composition and declamation a certain dignity and force added to the ideas which are conveyed. I may do the French tongue injustice, but it seems to me that if a French speaker is eloquent, it is rather in spite of his language, than from any aid which he derives therefrom. The language is suited, perhaps, better than the English, to nicety and sharpness in defining ideas, but if he wishes to be impressive he is obliged to call to his assistance gestures and even grimaces. The fact that so much action is habitually suited to the word in French conversation, as well as in public discourses, proves the correctness of the remark that I have made. Action is a necessary adjunct to the language, springing from its deficiency in intrinsic power. With us it is only a useful ally, often not essential, and very rarely admissible to the extent practiced in France. Webster and Clay, for example, needed no gesticulations to enchain the attention of their auditories, and overwhelm them with their eloquence. On the contrary, the effect of their simple but powerful diction would thereby have been impaired. Most of the lecturers that I have heard here, enunciate with a kind of musical intonation. During the pronunciation of a long sentence the voice is several times raised almost, or even quite to a *falsetto*, and finally drops to a low note at the conclusion of the sentence. But I am digressing in a way that I fear will be tedious.

You may be interested in some account of the substance of the lectures by M. Trousseau. The subbject of peritonitis has occupied a portion of his remarks, several cases having been under observation lately, in his wards. A patient was admitted with evidences of a slight degree of inflammation, but a considerable quantity of liquid effusion within the peritoneal cavity, which had taken place very rapidly. In the other cases the symptoms denoted more intense inflammation and the liquid effusion was small, the abdominal enlargement being due chiefly to meteorism. This point of difference led him to comment on the fact that quantity of effusion is no criterion of intensity of inflammation, but is determined by the *special* character of the inflammatory action.

In perionitis with effusion, he stated the risk from the operation of paracentesis to be much greater than in the case of other serous structures. In pleurisy, for example, the chest may be opened with greater impunity. In peritonitis it is very apt to be followed by exasperation of the disease and a fatal result.

For the treatment of peritonitis, he advocates calomel in fractional doses, viz., one grain taken in the course of the twenty-four hours, in ten or twelve doses, which will affect the gums in three or four days.

Opium and belladonna to relieve abdominal pains he deems highly important. On this point he dwelt at considerable length taking the view that the value of narcotics in painful inflammatory affections is not limited to their palliative effects. Pain, in itself, determines a fluxion to the painful part disposing to, and exasperating the state of inflammation. Thus in a person subject to gout, an attack may be determined by the pain incident to wearing a tight boot. Neuralgia seated in the supra-orbital nerve occasions an injection of the conjunctiva proportionate to the amount of pain. Let this pain be relieved by a local application of belladonna and opium, and the vascular injection ceases. The efficacy of opium in the treatment of peritonitis is no novelty on our side of the Atlantic. Indeed it is, I suspect, much better understood, with a portion of the American medical profession at least, than in Paris. I was surprised that M. TROUSSEAU did not mention as one of the important means by which its efficacy is exerted, arrest of the peristaltic intestinal movements.

He attached importance to local emollient and narcotic applications.

In a case of mild erysipelas seated on the face, he directed nothing but simple diet, and lemonade. This case furnishes a text for remarks on the tendency of certain inflammations to run a definite course, "*sans nous, malgré nous.*" Mild erysipelas of the face, excepting it be accompanied by accidental complications, or developed by epidemic influences, is rarely a grave affection, and does not claim active interference. So, frequently, phlegmonous inflammations will often run on to suppuration in spite of any measures, and if the latter have been energetic, the patient is only enfeebled by them, without having received any benefit. Tonsillitis was cited as an illustration. Frequently this affection is treated by bleeding, general and local, purging, vomiting, etc., with a view of arresting its progress. The object almost uniformly fails, and then, instead of recovering rapidly from the disease, the patient requires additional time to recruit from the effects of the treatment.

In another lecture he called attention to the case of an infant who presented, on auscultation of the chest, a well marked tubular respiration. He arrived at the diagnosis of tuberculous disease in view of the fact that in children under a year old true pneumonitis is extremely rare, in cases presenting the symptoms of pulmonary inflammation the affection being capillary bronchitis. Rapid phthisis in young children he stated to be as frequent as it is rare in adults.

In a case of paraplegia suddenly developed in association with the phenomena of the first stage of variola, he attributed the paralysis to the tonic effect of the variolous poison. On an autopsical examination of a case of the same description, the brain and spinal marrow were found perfectly healthy. The *sudden* accession of the paraplegia is evidence that it does not depend on softening, or other organic lesions of the spinal cord. The variolous poison in such a case exerts a special action on the nerves which is analogous to the operation of strychnia, veratria, and other similar agents.*

I have given these few points, from memory, merely to serve, in some degree as specimens of the matter of the clinical lectures by TROUSSEAU. I am not prepared nor could I spare sufficient time to undertake any thing like a full report of his lectures. Even if I had time, and sufficient familiarity with oral French to do this, a sufficient object is not to be found in the information

---

* The *Gazette des Hopitaux*, April 22, contains a brief report of several cases of paraplegia, incident to the development of small-pox. I send you a translation of the article.

which the lectures contain; for one conversant with current medical literature would not expect to derive from his oral instruction, thus reproduced, very much that is important and at the same time new; and although an able and highly interesting lecturer, I am by no means willing to admit that he is superior to several clinical teachers in our country.

The subject of small-pox, referred to just now, leads me to mention a fact which in my visits at different hospitals in Paris has struck me with surprise. At Hôtel Dieu, La Charité, St. Antoine, and Beaujon, I have noticed patients in the different stages of this disease intermingled with the other patients. No attempt at isolation is made. The effect of this is, that in the female ward under the charge of TROUSSEAU one of the patients just recovering from peritonitis is now attacked with varioloid. A patient with varioloid just being developed was received into a portion of the female ward devoted to children and their mothers. She remained there, no precautions being taken against infection, except to direct some young children who had never had vaccinia to be vaccinated. As a consequence, one of the children now has small-pox. It is truly remarkable that while such extensive, liberal hospital provisions exist, and so many institutions are devoted to special objects, a hospital exclusively for small-pox, and other contagious diseases, is not to be found in Paris.

Having, as you know, several years ago withdrawn permanently from the practice of surgery, my interest in that department is of course comparatively small, still I shall take pains to see the most distinguished of the Parisan surgeons. At *Hôtel Dieu* M. JOBERT (de Lambelle) is attached to the Surgical staff. I had often heard of him, and of his peculiar practice in uterine affections, and I was therefore desirous of witnessing his examination of patients and operations. I have devoted two mornings to this object. M. JOBERT is about fifty years of age; has dark hair and eyes, and his physiognomy is *distingué*. He is pompous in his carriage and mode of speaking, and *brusque* in his intercourse with his patients. One is led to notice these traits the more because they are in contrast with the manners of the other Surgeons here whom I have seen. In his ward visits on the two days when I was present, he examined the cases rapidly, and prescribed off hand, with a certain air of positiveness which might lead one to imagine that he had in view a moral effect upon the spectators as well as patients.

One of the days which I selected for visiting his wards was what is well known here as his *burning* day. Two days in the week, (Monday and Friday) are distinguished by this appellation. On these days he *burns* the os uteri in more or less of his cases. We have heard, on our side of the Atlantic, cauterization with the nitrate of silver stigmatized as "womb-burning," but in the practice of M. Jobert this expression has a literal signification. He introduces into the vagina, through the speculum, an iron heated red hot, and brings it into contact with the mouth of the uterus. On the two days of the week just mentioned, after making his visit in the wards he received patients requiring examination with the speculum in a small room devoted to that purpose. In addition to the *internes* and *externes* about a dozen students and physicians were present on the Friday which I selected for the day of my visit. With this number the room was quite crowded. The females were arranged in a row in the hall, admitted one by one, placed on a table, the limbs separated and held by an *interne* on each side, the organs exposed to the light from a window opposite, and but a few feet distant from the patient. About twenty patients were examined. M. Jobert employed different specula, the cylindrical, the bivalve, and in some instances several blades or spatulæ, introduced separately. He applied the hot iron on this occasion in two cases. In one case the mouth and neck of the uterus, before the application, was swelled, reddened, and presented superficial ulcerations. He allowed the iron to remain in contact, I should judge, nearly half a minute. It gave rise to a hissing sound, considerable smoke, and the characteristic smell of burning animal matter. It recalled from the dimmed memory of the classical studies of youth the story of the Cyclops; yet the application did not appear to occasion suffering. The patient did not shrink, nor utter any expressions of pain. In the other instance there was extensive destruction of the uterus from ulceration. In this case the application was apparently unattended by pain. The woman complained when the speculum was introduced but not while the uterus was burning.

Of the effects of this method of treatment, in these cases, I cannot speak from observation, as I have not been present at any of the subsequent *burning* days. I am not aware that the practice has been adopted by any other of the Parisian surgeons, a fact certainly not in its favor, but not sufficient perhaps to disprove its propriety.

In one of the patients examined by M. JOBERT there was complete extrusion of the uterus, forming a tumor between the thighs of the size of a coffee cup. The bladder was dragged down and connected with the upper part of the tumor, and there existed also considerable prolapsus of the rectum. M. JOBERT introduced a sound into the uterus, and also a catheter into the bladder to show its situation. In the latter operation the instrument required to be passed in a direction downward and outward. The patient was dismissed without any treatment.

In my last letter I stated that Epidemic Cholera had ceased to exist at Paris. This was based on a statement to that effect in the *Gazette des Hopitaux.* The statement proved to be erroneous. Cases have been admitted into the several hospitals up to the present time. At La Charité some cases have been developed within the wards. The number of cases that have occurred is however small, and is diminishing. No alarm is felt in the city, nor does there appear to be that general tendency to diarrhœa which accompanies this disease when it prevails to much extent. So far as I can judge from the hospitals, the city is healthy.

You will recollect that the subject of artificial syphilization, as a mode of protecting the system against the syphilitic virus, was agitated here a few months since. The subject is now only referred to as one of the curious by-gone medical novelties that have produced a transient excitement. The practice was condemned by the Academy of Medicine, and interdicted by the Prefect of the Seine. I am informed that its author, M. AUZIAS TURENNE, has admitted the error of the doctrine, which, if true, is some thing in palliation of having prematurely advocated it. A medical friend stated to me that much injury was done by his experiments at the hospital LOURCINE, and that a young Prosector of Anatomy who had submitted to experimental inoculations for syphilis, is now laboring under constitutional effects of the disease thus induced, which resist medical treatment, and will be likely to ruin his health permanently.

An instance of sudden death from the effects of Chloroform has lately occurred at the hospital St. Antoine. The following is a statement of the facts given by the operating Surgeon M. Richard, to the *Societé de Chirurgie* on the day that the event took place:

"The patient entered the ward of M. Richard to have removed a polypus of the uterus. The polypus was fibrous, of the size of a small apple, with a peduncle of the size of the little finger, sufficiently long, attached to the middle of the anterior face of the uterine cavity. The tumor was reached, without difficulty, and easily brought down for the operation. The patient presented the ordinary symptoms, except that the hemorrhage had been but moderate, and, although somewhat pallid, she had preserved her strength and the conditions of health as regards sleep and appetite. I decided to cut off the polypus, and to touch the surface to which it was attached with the perchloride of iron, a practice which, lately, in a similar case, had been attended with prompt and complete success.

"There was in my view a double contra-indication in the case, for the use of chloroform. First, the slight pain of the operation, and second, the sub-anæmic state of the patient. This fact I presented to her at each of my morning visits, and I even entreated her to consent that I should operate without chloroform; but the poor woman declared that it must be impossible to remove a tumor from within the body without great suffering, and at any rate she would not be operated on unless she was put to sleep. My opposition yielded to hers, having so often employed, and witnessed the employment of chloroform in cases which much less than this one demanded its use.

"At nine o'clock this morning, everything being prepared for the operation, I proceeded to induce anæsthetization. The woman rested on the bed; and I permitted her to lie without disturbing her, intending to place her in a proper position at the moment of the operation. I simply removed a pillow, in order that the head might be on the plane of the rest of the body, I said to her "look at me and breathe as I do," and I then imitated the respirations of a person sleeping quietly. I poured a certain quantity of chloroform upon a compress of five or six thicknesses, and placed it before the face at a certain distance in order that the vapor of the chloroform might be well mixed with common air. I administered the chloroform myself up to the moment when the position of the patient was changed, and I may here state to the Society that I have always pursued this course. For two years I have often been placed at the head of an active surgical service, particularly at the St. Louis hospital, and, so far as my memory serves me, I have never failed myself to produce the anæsthesia up to the moment when it was necessary to take the bistoury.

"The respiration and the pulse were regular for about a minute. Two or three times I added a certain quantity of the anæsthetic liquid, the precise quantity not determinable, the administration being conducted in the way it is daily done by every one. At the end of a minute there was agitation. I abandoned the compress to see if I could not operate upon the patient lying on the bed, without altering her position. The agitation increased, she struggled so as to require to be held by several assistants, and talked incoherently. The compress which I had entrusted to an *élève*, by my direction was removed, and soon replaced with a fresh dose of chloroform. From this moment the agitation ceased. I ordered the patient to be turned so that the limbs might be placed out of the bed. This second period, commencing with the agitation, lasted from a half minute to a minute at most. I had introduced my hand into the vagina and drawn out the polypus, to the peduncle of which a ligature was attached. About two minutes had elapsed from the commencement of the administration of the chloroform, and the compress had been withdrawn for some seconds, I felt for the pulse and found none. I let go the polypus, and it was drawn back. The respiration continued, but slowly; the face became cadaveric, and the eye lustreless. Quickly the head was lowered and the legs and arms raised. The face resumed a little color; respirations continued, but became more and more infrequent. I then produced artificial respiration by making rhythmical pressure on the chest. At the same time the assistants resorted to blows on the thighs, the calves of the legs, the arms, and friction on the face. Several times we titillated the top of the larynx. All these efforts were unavailing. On suspending the artificial respiration there were two or three long inspirations, after long intervals, and then cessation. Coldness became general but the beating of the heart was still heard, although feeble, by one of the *internes*. These efforts were continued for ten minutes. My two colleagues, M. Aran and M. Herard, entered just as I had opened the trachea. I practiced insufflation through the orifice while some one continued the artificial respiration. M. Herard introduced into the precordial region two needles communicating with a powerful galvanic battery. The thoracic muscles were violently convulsed, but no pulse was developed. At length we ceased further exertions after half an hour's duration of this terrible contest.

"In order that this deplorable misfortune may prove as instructive as possible, I will invite any member to propose questions, in case some details may have escaped me."

M. Gosselin who made the autopsy in the presence of M. M. Richet, Debont and Marjolin, reported as followes: No marked appreciable lesions were discovered. The lungs were healthy excepting a little sub-pleural emphysema, without ecchymosis. The heart was flaccid and empty. This softness was especially marked in the walls of the right cavities. There was but little blood in the cavities. Coagulated blood was nowhere found; it preserved its fluidity everywhere. There was no encephalic congestion. In the superficial veins of the head there were some bubbles of gas, but this was due to cadaveric decomposition. In a body which we opened immediately, in the amphitheatre, we found the same development of gas. In the veins of the liver, also, there was a little gas.

To sum up, so far as we could judge, the autopsy did not bring into view any cause of death. We removed the blood found in the cavities of the heart, placing in separate bottles that taken from the right and the left side. The blood did not present any of the odor of chloroform.

M. Debout stated that an analysis of the blood demonstrated the presence of a very minute quantity of chloroform.*

---

* The foregoing account is from the *Gazette des Hopitaux*.

### ON PARAPLEGIA DUE TO VARIOLA AND OTHER CAUSES INDEPENDENT OF A PERSISTENT LESION OF THE SPINAL CORD.

*Translated from the Gazette des Hopitaux; April* 22, 1854.*

In connection with facts pertaining to paralysis symptomatic of cerebral lesions, of which we have lately reported several instances, it will be of interest to present facts relating to paralysis either idiopathic or symptomatic, but occurring independently of any appreciable persisting lesion of the nervous centres. Illustrations of the latter kind are frequently met with, especially in cases of paraplegia. It is very rare to find examples in cases of hemiplegia, although they occasionally occur, and but a short time since, we reported a remarkable instance. The facts to which we propose to devote a brief space, and which have lately fallen under observation, relate to paraplegia, and some of them to a species of paraplegia, respecting which authors in general are silent—we refer to paraplegia due to variola.

The first instance of the latter, to which our attention has been called by M. TROUSSEAU, was in a patient, of whom we have already given our readers some account. A young female who entered the hospital for a *hydro-peritonitis*, and who had been treated with calomel, was improving, when she was attacked with fever, pains in the loins, and vomiting. Under the impression, at first, that the pain and febrile movement were owing to an exacerbation of the peritoneal inflammation, M. TROUSSEAU prescribed again calomel. But the fever persisting, and becoming more intense the following day, without any augmentation of abdominal pain and tenderness, it became necessary to abandon this first impression. On the other hand, the pains in the loins had become exceedingly acute, and on farther examination it was a matter of not a little surprise to find that the inferior extremities were para-

* This article is referred to in the second letter from Paris written by the Translator.

lyzed. They were absolutely powerless, and the sensibility also was considerably diminished.

Taking into consideration the existence of several cases of small-pox in the ward, and recollecting that he had seen, several times, the eruption of variola preceded by a transient paraplegia, M. Trousseau at once diagnosed, in the case of this young female, a paraplegia due to an *intoxication variolique*, and predicted that the eruption would shortly appear. It was not long before this prediction was fulfilled. On the day following a characteristic eruption covered the face, the chest, and portions of the extremeties.*

A short time before, they who are accustomed to follow M. Trousseau in his visits had seen a young female with a paraplegia of the same description, which continued only during the prodromic stage of variola, disappearing the moment the eruption took place. By a singular coincidence there occurred, at the same time, a case precisely similar in the service of M. Rostan. A young female entered with fever, acute pain in the loins, and paraplegia. Twenty cups were ordered. The next day the paraplegia did not exist, but well marked variola.

At this moment, in the ward appropriated to nursing women, there is a woman, whose infant died with cholera, who has also had variola preceded by paraplegia.

It should be remarked that in all such cases the paraplegia does not continue except during the prodromis period, or at most, during the continuance of the variola. Generally the patients recover entirely the use of the limbs after the complete evolution of the eruption.

In the same ward (St. Bernard) there is a woman affected with idiopathic paraplegia, which does not appear to be connected, either symptomatically, or sympathetically, with any lesion, or other affection. This woman entered the hospital for pulmonary catarrh. Afterward, having been cured of her pulmonary catarrh, she was seized with catarrh of the intestinal canal, of which she was relieved by a few pills of the nitrate of silver. She was convalescent, and on the point of leaving the hospital, when she

* This case was seen by the Translator.

found her lower extremities paralyzed, the paralysis confined to motion, sensibility being preserved. M. TROUSSEAU prescribed thirty wet cups, by which were taken away about five ounces (by weight) of blood. The next day the paraplegia had disappeared. The patient was cured.

An instance quite similar to the latter presented itself during the past year in the service of M. TROUSSEAU. A man was admitted for rheumatism affecting the shoulder. He was treated with belladonna. The rheumatism left the shoulder and fixed itself in the cervical plexus, from which followed paralysis at the arm, affecting both motion and sensation. Satisfied that this paralysis was due to a rheumatic affection, and that it did not involve a cerebral lesion, M. TROUSSEAU continued the same medication, and in a short time both the rheumatism and paralysis disappeared. This man stated that he had had an attack of paralysis after taking a cold bath while heated, and that the paralysis in a short time disappeared of its own accord.

The inference from these facts, to which might be added many others of a similar character, is, that we must not be in haste to form a grave prognosis in cases of paraplegia, or of paralysis of different parts of the body, which often depend on slight causes; nor should we hasten to oppose a medication active, energetic or painful to an affection which may disappear of itself in a short time.

